# HSP70 is a negative regulator of NLRP3 inflammasome activation

**DOI:** 10.1038/s41419-019-1491-7

**Published:** 2019-03-15

**Authors:** Pierre Martine, Angélique Chevriaux, Valentin Derangère, Lionel Apetoh, Carmen Garrido, François Ghiringhelli, Cédric Rébé

**Affiliations:** 1 INSERM UMR1231, F-21000 Dijon, France; 2 Université Bourgogne Franche-Comté, F-21000 Dijon, France; 3 Centre Georges François Leclerc, F-21000 Dijon, France

## Abstract

The NOD-leucine rich repeat and pyrin containing protein 3 (NLRP3) inflammasome is a multi-protein complex, aimed at producing IL-1β in response to danger signals which must be tightly regulated. Here we investigated the importance of the stress sensor, Heat Shock Protein 70 (HSP70) on NLRP3 inflammasome activation. HSP70 deficiency leads to the worsening of NLRP3-dependent peritonitis in mice. HSP70 deficiency also enhances caspase-1 activation and IL-1β production in murine Bone Marrow-Derived Macrophages (BMDMs) under NLRP3 activator treatment in vitro. This observation is associated with an increased number and size of Apoptosis associated Speck-like protein containing a CARD domain (ASC)/NLRP3 specks. Conversely, the overexpression of HSP70 in BMDMs decreases caspase-1 activation and IL-1β production under NLRP3 activator treatment. HSP70 interacts with NLRP3 and this interaction is lost upon NLRP3 inflammasome activation. Heat shock inhibits NLRP3 inflammasome activation in vitro and inhibits peritonitis in mice. Therefore this study provides evidence on the inhibitory role of HSP70 on NLRP3 inflammasome and open the possibility of treating inflammatory diseases via HSP70 induction and/or by hyperthermia.

## Introduction

Inflammasomes are intracellular complexes constituted by a receptor and an adaptor that enable recruitment and activation of pro-inflammatory caspases such as caspase-1 and the maturation of pro-inflammatory cytokines such as IL-1β or IL-18^1^. The NOD-leucine rich repeat containing proteins (NLR) are activated by a wide diversity of stimuli called PAMPs (*pathogen-associated molecular patterns*) or DAMPs (danger-associated molecular patterns) or environmental stresses. Specific domains characterize NLR family. The central NACHT domain is responsible for ATP-dependent oligomerization whereas the C-terminal LRR (Leucine Reach Repeat) domain is implicated in the detection of activating signals and in complex autoregulation. These receptors contain, on the N-terminal side, a CARD (CAspase Recruitment Domain) or a PYD (PYrin Domain) implicated in protein/protein interactions to transduce the signal. Then, activated NLRs can recruit either pro-caspases or adaptors proteins (via the PYD) that will in turn recruit pro-caspases^2^.

NLRP3 inflammasome is the most widely described complex. It consists of NLRP3, the adaptor ASC (Apoptosis associated Speck-like protein containing a CARD domain) and pro-caspase-1. In the absence of any stimuli, NLRP3 is maintained in an inactive form at the endoplasmic reticulum level. ASC is mainly localized at the mitochondria level. Misawa et al. suggested that upon activation, the intracellular concentration of NAD^+^ decreases, leading to the inactivation of SIRT2 (sirtuin 2) and the accumulation of acetylated α-tubulin responsible for the approach between mitochondria and ER^3^. This enables the interaction of ASC with NLRP3, through the PYD and the polymerization of ASC into filaments^4^. This oligomerized complex can recruit pro-caspase-1 via the CARDs, leading to the cleavage and activation of pro-caspase-1. The active caspase-1 will in turn cleave pro-IL-1β and pro-IL-18 to produce mature IL-1β and IL-18^3^.

NLRP3 inflammasome is activated in two different steps, priming and activation. Priming step begins with the recognition by PRRs (pattern recognition receptors) of extracellular molecules such as LPS (LipoPolySaccharides), TNFα (Tumor Necrosis Factor  α) or IL-1β^5^. This will have two consequences: the activation of NF-κB (Nuclear Factor-kappa B) leading to NLRP3 and pro-IL-1β transcription^6^ and deubiquitinylation of the LRR domain of NLRP3, required for its activation^7,8^. The activation step is engaged when cells are exposed to endogenous or exogenous molecules such as ATP, via P2X7 receptors or the bacterial toxin nigericin, that both induce a decrease in intracellular potassium concentrations or the phagocytosis of crystal structures, such as MSU (monosodium urate)^9–11^. Once constituted, NLRP3 inflammasome is then secreted into cell supernatant and can amplify the inflammatory response by activating the inflammasome and caspase-1 in neighbouring cells^12,13^.

IL-1β is mainly produced by intracellular protein platforms inflammasomes, especially in patients presenting gain-of-function mutations in genes coding for these complexes constituents, e.g., NLRP3 and suffering from periodic fever syndromes, such as cryopyrin associated periodic syndromes^14^. IL-1β is one of the most important mediators leading to fever, a very frequent symptom of these diseases, so that it is called the ‘pyrogenic cytokine'^15^. However, even if inflammation is well-known to induce fever, little is known about the effects of fever on inflammation. The most important cellular mediators involved in hyperthermia effects are the Heat Shock Proteins (HSPs).

HSP family participates in the cellular response to environmental or pathophysiological stress conditions. These proteins also control initiator caspases activation in platform complexes such as the DISC (Death Inducing Signaling Complex) or the Apoptosome^16^. Because inflammasomes are also platforms that enable activation of inflammatory caspases, one can speculate that HSPs might also control these complexes. Thus, HSP90 has been shown to regulate the inflammasome activation^17^. However the importance of other HSPs (especially inducible HSPs) on this complex has yet to be determined. In this study we focused on the inducible HSP70. We show that the lack of HSP70 is associated with an over-activation of NLRP3 inflammasome and a more important activation of caspase-1 and maturation of IL-1β. Conversely, HSP70 overexpression or a heat shock, impair these events. Our work thus establishes HSP70 as a new negative regulator of NLRP3 inflammasome activation.

## Materials and methods

### Reagents

LPS (L3024), ATP (A7699) and nigericin (N7143) were purchased from Sigma-Aldrich (St. Louis, MO, USA). MSU (tlr-msu) was purchased from Invivogen (San Diego, CA, USA). Alum (77161) was purchased from Thermo-Scientific (Thermo Fisher Scientific, MA, USA). M-CSF (216-MC) was purchased from R&D Systems (Lille, France). Recombinant HSP70 was kindly provided by Carmen Garrido’s team.

### Cell culture

Human myeloid THP-1 cells were obtained from the American Type Culture Collection (ATCC—Manassas, VA, USA) and were grown in RPMI 1640 with ultraglutamine (Lonza, Basel, Switzerland) supplemented with 10% (vol/vol) fetal bovine serum (FBS; Lonza) and with Pen/Strep Amphotericin B (PSA, Lonza) 1%, in an atmosphere of 95% air and 5% CO_2_ at 37 °C. Prior to the experiments THP-1 cells were primed with LPS (10 ng/mL, Sigma-Aldrich) for 20 h. In some experiments a heat shock was performed by incubating the cells at 42 °C for 1 h. Cells were then left at 37 °C for 2 h.

### Mice

All animals were bred and maintained according to both the FELASA and the Animal Experimental Ethics Committee Guidelines (University of Burgundy, France). Animals were used between 6 and 22 weeks of age. Female C57BL/6 mice (aged 6–8 weeks) were obtained from Charles River Laboratories (Saint Germain sur l’Arbresle, France). C57BL/6 HSP70^−/−^ mice were generated by a 12-kb deletion of both HSP70.1 and HSP70.3-coding regions^18^ and were obtained from the Mutant Mouse Resource and Research Center (MMRRC), bred and maintained in the Cryopréservation, Distribution, Typage et Archivage Animal (CDTA-Orléans, France).

### In vivo experiments

For the hyperthermia experiments mice were placed on a heating pad at 42 °C for 1 h and were allowed to rest overnight^19^. Mice were then treated by IP injection of either Alum (200 µL, 40 mg/mL) or MSU (200 µL, 10 mg/mL) for 4 h. A peritoneal lavage with 5 mL of PBS was performed to collect cells and supernatants^20^. All the data, corresponding to five animals per group, were collected along different experiments.

### Flow cytometry experiments

Collected peritoneal fluids were centrifuged to pellet cells. The total number of cells was evaluated and a part of these cells were stained with a PE rat anti-mouse Gr1 (551461) and an APC rat anti-mouse CD11b (553312) (BD Bioscience, Le Pont de Claix, France) for 20 min at 4 °C. After washing, cells were analyzed with an LSRII flow cytometer (BD Biosciences) and analyzed using Flowjo software.

### Mouse bone marrow-derived macrophages

C57BL/6 mice bone marrow cells were isolated from tibias and femurs and cultured for 6 days on plastic plates in DMEM high glucose medium with ultraglutamine (Lonza) supplemented with 10% (vol/vol) fetal bovine serum (FBS; Lonza) in the presence of 50 ng/mL of M-CSF, in an atmosphere of 95% air and 5% CO_2_ at 37 °C. Subsequently, floating cells were removed and macrophage differentiation was observed by fibroblast-like shape changes visualized with a Zeiss PrimoVert microscope. Differentiated cells were then primed with LPS (100 ng/mL—Sigma-Aldrich) for 20 h and treated in OptiMEM by different inflammasome activators: ATP (5 mM) or Nigericin (40 µM) for 30 min, MSU (100 µg/mL) or Alum (100 µg/mL) for 6 h.

### IL-1β detection

Murine IL-1β was detected using the Mouse IL-1 beta/IL-1F2 DuoSet ELISA (DY401-05) kit from R&D Systems according to manufacturer’s instruction. Briefly, 96-wells plates were coated overnight at room temperature with 100 μL of diluted IL-1β capture antibody at 4 µg/mL. After washing three times, wells were blocked for 1 h. Then, 100 µL of samples or standards were incubated for 2 h at room temperature. After additional three washes, 100 µL of diluted detection antibody at 500 ng/mL was added at room temperature for 2 h. Detection was performed using streptavidin-coupled HRP and its substrate with a microplate reader set at 450 nm. Concentration was evaluated using a standard curve.

Human IL-1β was detected using the HEK-Blue™ IL-1β cells (Invivogen) according to manufacturer’s instructions.

### Supernatant precipitation

LPS-primed cells (1.5 × 10^6^/500 µL) were treated in OptiMEM without FBS. The supernatants were harvested by centrifugation for 5 min. at 400 × *g* and precipitated using methanol (500 µL) and chloroform (150 µL). After centrifugation at maximum speed for 10 min., the aqueous phase (at the top) was discarded and 800 µL of methanol were added. Samples were centrifuged at maximum speed for 10 min and the supernatants were removed. Pellets (containing proteins) were dried for 10 min. at 37 °C, mixed with 40 µL of loading buffer (125 mM Tris-HCl [pH 6.8], 10% β-mercaptoethanol, 4.6% SDS, 20% glycerol, and 0.003% bromophenol blue) and incubated at 95 °C for 5 min.

### Western blotting

Whole-cell lysates were prepared by lysing cells in boiling buffer (1% SDS, 1 mM sodium vanadate, 10 mM Tris [pH 7.4]) in the presence of complete protease inhibitor mixture. Samples viscosity was reduced by sonication.

Whole-cell lysates or immunoprecipitated samples were mixed with loading buffer and separated by sodium dodecyl sulfate–polyacrylamide gel electrophoresis (SDS-PAGE), and electroblotted to a nitrocellulose membrane (Amersham, GE Healthcare, Velizy-Villacoublay, France). After incubation for 1 h at RT with 5% nonfat milk in phosphate-buffered saline (PBS)–0.1% Tween-20, membranes were incubated overnight with the primary antibody diluted in PBS-milk-Tween, washed, incubated with the secondary antibody for 30 min at RT, and washed again before analysis with a chemiluminescence detection kit (Amersham, GE Healthcare). The following mouse mAbs were used: anti–β-actin (A1978) from Sigma-Aldrich, anti-NLRP3 (AG-20B-0014), anti-human caspase-1 (AG-20B-0048) and anti-murine caspase-1 (AG-20B-0044) from Adipogen (COGER SAS, Paris, France). Rat pAbs anti-IL-1β (401-M) from R&D Systems and rabbit pAbs anti-ASC (AL177), anti-HSP27 (SPA-803), anti-HSP70 (SPA-812) and anti-HSP90 (SPS-711) from Enzo life sciences (Villeurbanne, France) were also used. Secondary Abs HRP-conjugated polyclonal goat anti-mouse and swine anti-rabbit immunoglobulins (Jackson ImmunoResearch, Interchim, Montluçon, France) were used.

### Immunoprecipitations

Untreated or nigericin-treated cells (50.10^6^) were lysed in 1 mL lysis buffer (25 mM Hepes (pH8), 150 mM NaCl, 0.5% Triton X-100, 5 mM EDTA, 10% glycerol, 1 mM NaVO_4_, 20 mM NaF and CPIM) for 30 min on ice. After centrifugation at 14,000 × *g* at 4 °C for 30 min, supernatants were precleared during 2 h at 4 °C in the presence of 30 μL of mixed Sepharose 6B (6B100, Sigma-Aldrich) and protein G (17-0618-01, Amersham, GE Healthcare). After centrifugation at 1000 g for 3 min the supernatant was incubated with 1 μg/mL of anti-HSP70 antibody (ADI-SPA-812, Enzo life sciences) and 40 μL of mixed Sepharose at 4 °C for 20 h. The precipitates were washed four times in lysis buffer, eluted in loading buffer and analyzed by immunoblotting.

### In vitro caspase-1 activation assay

LPS-primed (or not) THP-1 cells were pelleted and incubated in three volumes of hypotonic buffer (20 mM HEPES-KOH [pH 7.5], 10 mM KCl, 1.5 mM MgCl_2_, 1 mM Na EDTA, 1 mM Na EGTA and CPIM) for 15 min. on ice. Cellular membrane integrity was disrupted by passage through a G22 needle. Cell lysates were centrifuged at maximum speed for 20 min. at 4 °C and supernatants were harvested. Inflammasome activation was induced by incubating 120 µg of the previously obtained extracts in a final volume of 100 µL at 37 °C for 30 min and caspase-1 cleavage was monitored by Western blot.

### Immunofluorescence (IF) and in situ proximity ligation assay (PLA)

Cells (150,000) were seeded in 12 well-dishes containing a cover glass (631–0150, VWR International, Fontenay-sous-Bois, France) which was pretreated for 10 min with Poly-L-Lysin (P4707, Sigma Aldrich). The following day, cells were treated with LPS at 100 ng/mL for 20 h and then with different inflammasome activators. Cells were washed in PBS, fixed with 4% PFA at 4 °C for 10 min and permeabilized using a PBS, 0.5% BSA, 0.1% Saponin (47036, Sigma Aldrich) buffer for 20 min at RT. Samples were incubated 2 h at RT with primary antibodies or with Ig as a control.

For IF experiments, cells were washed two times, and incubated with secondary Alexa488 or Alexa568 conjugated anti-mouse or anti-rabbit for 30 min at RT. For PLA experiments (Sigma-Aldrich DUO92007), after washing primary antibodies, cells were then incubated with the appropriate probes (Sigma Aldrich DUO92004 and DUO92002) during one hour at 37 °C and washed two times. Probes were then ligated for 30 min at 37 °C, washed two times in Buffer A and amplified using the manufacturer’s polymerase for 100 min at 37 °C in the dark.

For both experiments, cover glasses were mounted on a drop of Mounting Medium containing Dapi (Duo82040, Sigma Aldrich) for 15 min in the dark on a microscopy slide (045796, Dutscher, Brumath, France). Slides were imaged using a CDD equipped upright microscope (Zeiss) and ×63, 1.4NA objective. Image analysis was performed using ImageJ software.

The following antibodies were used for IF and PLA: mouse anti-NLRP3 (1/500, AG-20B-0014, Adipogen), and anti-caspase-1 (AG-20B-0044, Adipogen), rabbit anti-ASC (1/1000, AL-177, Enzo Life Science), anti-HSP70 (1/200, AL-812, Enzo Life Science) goat anti-mouse Alexa488 (1/1000, A11029, Invitrogen), goat anti-rabbit Alexa568 (1/1000, A11036, Invitrogen, Life Technologies, Villebon sur Yvette, France), donkey anti-mouse Alexa568 (1/1000, A10037, Invitrogen).

### Transient transfections

For the siRNA experiments, BMDMs were transfected with the INTERFERin™ transfection reagent (Polyplus transfection, Illkirch, France) according to manufacturer’s instructions. Briefly, 5.10^6^ cells were seeded in 145 mm petri dishes one day before transfection. Silencer® Select siRNAs specific for the target gene or Negative siRNA control (Life Technologies) diluted in serum-free medium were incubated with INTERFERin™ for 10 min at room temperature and added to the cells to a final concentration of 1 nM. Twenty four hours after transfection, transfection medium was removed and the cells were incubated with fresh medium. The following siRNA were used: Hspa1a (s101440), Hspa1b (s201488) or control (AM4611).

For overexpression experiments, BMDMs were transfected with the Lipofectamine 2000 transfection reagent (Thermo Fisher Scientific) according to manufacturer instructions. Five million cells were seeded in 145 mm petri dishes one day before transfections. The plasmids were diluted in serum-free medium and were incubated with Lipofectamine 2000 reagent for 20 min at room temperature and added to the cells at a final concentration of 2.5 µg/mL. Twenty four hours after transfection, the transfection medium was removed and the cells were incubated with fresh medium. HA-PCDNA3 empty vector and HA-PCDNA3-HSP70 were kindly provided by Aurélie DeThonel.

### Statistical analyses

In vitro results are shown as means ± s.d., in vivo results are shown as means ± s.e.m. and comparisons of datasets were performed using unpaired Student’s *t* test (test group compared to control group). Statistical calculations were performed using GraphPad Prism 5. All *P* values were two tailed.

## Results

### Hsp70 deficiency increases caspase-1 activation and IL-1β maturation in vivo and in vitro

**Fig. 1:**
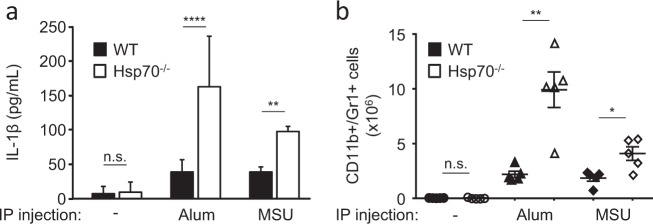
HSP70 deficiency increases peritonitis in mice. C57BL/6 WT or Hsp70^−/−^ mice (5 animals per group) were intraperitoneally injected with Alum (40 mg/mL) or MSU (10 mg/mL) for 6 h. IL-1β content in the lavage fluid was measured by ELISA (**a**) and the absolute number of neutrophils (CD11b/Gr1+) recruited was evaluated by flow cytometry (**b**). Data represents the mean ± s.e.m. of three independent experiments. Statistics compare WT and Hsp70^−/−^ animals: *p < 0.05; **p < 0.01; ****p < 0.0001; *n.s*. not significant using two tailed *t* test

**Fig. 2:**
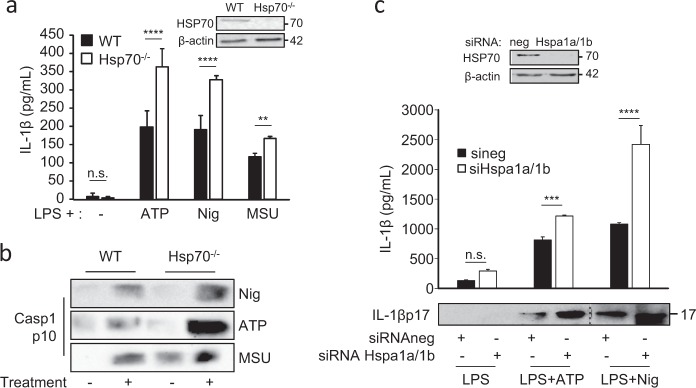
HSP70 deficiency increases caspase-1 activation and IL-1β maturation. BMDMs from WT or Hsp70^−/−^ C57BL/6 mice were stimulated with 100 ng/mL LPS for 20 h and then left untreated or treated 30 min with ATP (5 mM) or nigericin (Nig, 40 µM) or 6 h with MSU (100 µg/mL). **a** Cell lysates were immunoblotted to detect HSP70 expression (inset). Supernatants were collected and IL-1β was measured by ELISA (**a**) and caspase-1 p10 cleavage fragment was detected by western blot (**b**). **c** BMDMs from WT C57BL/6 mice were transfected with control siRNA or Hspa1a + Hspa1b siRNA, stimulated with 100 ng/mL LPS for 20 h and then left untreated or treated 30 min with ATP (5 mM) or nigericin (Nig, 40 µM). Cell lysates were immunoblotted to detect HSP70 expression (inset). β-Actin was used as a loading control. IL-1β was measured by ELISA or western blot in the supernatants. Data are the mean of at least three independent experiments ± s.d. Statistics compare WT and Hsp70^−/−^ BMDMs: **p < 0.01; ***p < 0.001; ****p < 0.0001, *n.s*. not significant using two tailed *t* test

To evaluate the impact of HSP70 on NLRP3 inflammasome activation, we used in vivo crystal-induced peritonitis models^20^. Wild type (WT) or Hsp70^−/−^ mice were intraperitoneally injected with aluminum salts (Alum) or monosodium urate (MSU). As previously described, Alum and MSU induced the recruitment of neutrophils and the maturation of IL-1β in the peritoneum of WT mice. These observations were exacerbated in Hsp70^−/−^ mice (Figs. 1a, b). To study the impact of HSP70 deficiency in vitro, BMDMs (Bone Marrow-Derived Macrophages) from WT and Hsp70^−/−^ mice were generated and primed with LPS. We checked the deficiency of HSP70 in HSP70^−/−^ BMDMs and the expression of other HSPs and of NLRP3 inflammasome main constituents (Supplementary Figure 1). After treatment with inflammasome activators ATP, nigericin or MSU, BMDMs from Hsp70^−/−^ mice were more effective at producing and releasing active caspase-1 and IL-1β in the supernatant than WT BMDMs (Figs. 2a, b and supplementary Figure 2). As a control, the production of TNFα remained unchanged (Supplementary Figure 3). These differences cannot be explained by a more important sensitivity of HSP70^−/−^ cells towards inflammasome activators cytotoxicity as ATP, nigericin and Alum decreased similarly WT and HSP70^−/−^ BMDMs viability (Supplementary Figure 4). To confirm the importance of HSP70, we used siRNA specific for Hspa1a and Hspa1b as an alternative strategy to knock-down Hsp70 in murine BMDMs. Again, we found that HSP70 depletion emphasized IL-1β maturation in supernatants of BMDMs treated with ATP or nigericin (Fig. 2c). Altogether these results suggest that HSP70 deficiency specifically emphasizes caspase-1 activation and IL-1β maturation.

### Hsp70 deficiency regulates NLRP3/ASC speck formation

**Fig. 3:**
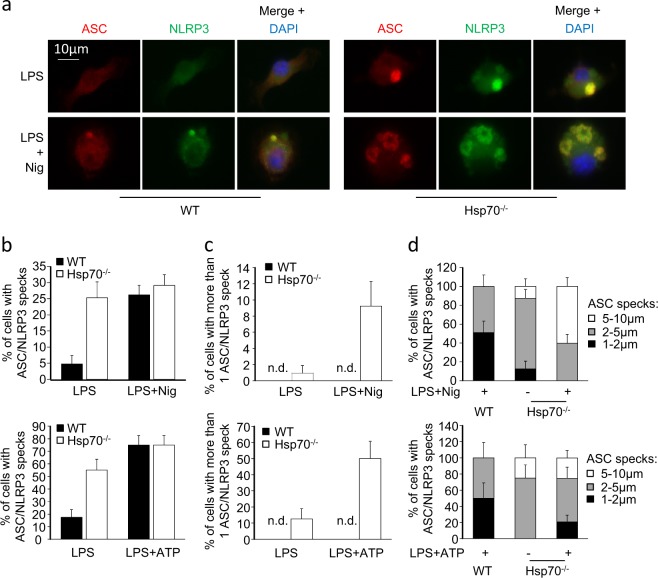
HSP70 deficiency increases NLRP3/ASC speck formation. BMDMs from WT or Hsp70^−/−^ C57BL/6 mice were stimulated with 100 ng/mL LPS for 20 h and then left untreated or treated 30 min with ATP (5 mM) or nigericin (Nig, 40 µM). Cells were stained with ASC and NLRP3 antibodies and with DAPI. Representative images are shown (**a**). The proportion of cells with ASC/NLRP3 specks (**b**), with more than one speck (**c**) and the repartition of cells according to the speck size within positive cells (**d**) were evaluated. *n.d.* not detected. Data are the mean of at least three independent experiments ± s.d

NLRP3 activation may occur through two distinct steps: the priming and the activation signals^5^. To investigate the impact of HSP70 on the priming step, mRNA expression of *Nlrp3* and *Il1b* was evaluated in WT and Hsp70^−/−^ BMDMs under LPS treatment. No differences were observed in *Nlrp3* and *Il1b* induction by LPS (Supplementary Figure 5). Caspase-1 activation also requires the oligomerization of NLRP3 which serves as a scaffold to nucleate ASC and to form ‘ASC-specks' or ‘ASC pyroptosomes'^1,21^. In murine WT or Hsp70^−/−^ BMDMs, nigericin or ATP induced the formation of ASC/NLRP3 specks as assessed by immunofluorescence staining (Figs. 3a, b). In Hsp70^−/−^ BMDMs, crude LPS was sufficient to induce ASC/NLRP3 speck formation whereas this was not possible in WT cells. When Hsp70^−/−^ BMDMs were treated with nigericin or ATP, we observed an increase of the number of specks per cell (Figs. 3a, c). Moreover, the size of ASC/NLRP3 specks was also modified. Resting Hsp70^−/−^ BMDMs presented a bigger ASC speck (2–5 µm) than nigericin or ATP-treated WT cells (1–2 µm). Finally, nigericin treated Hsp70^−/−^ BMDMs presented huge specks, reaching 5–10 µm (Figs. 3a, d). These results were confirmed by ASC/procaspase-1 oligomers detection (Supplementary Figure 6). The impact of HSP70 deletion on other inflammasomes was evaluated. When BMDMs were infected with LPS to induce non-canonical inflammasome activation, there was no difference in caspase-1 activation and IL-1β maturation between WT and Hsp70^−/−^ cells (Supplementary Figure 7). When cells were treated with the AIM inflammasome activator, poly(dA:dT), ASC specks were more abundant in WT BMDMs than in Hsp70^−/−^ BMDMs (Supplementary Figure 7). These findings suggest that the caspase-1 hyperactivation in Hsp70^−/−^ cells is mediated by an increased formation of NLRP3/ASC specks and that HSP70 seems to have no effect on other inflammasomes.

### HSP70 inhibits NLRP3 inflammasome activation by direct interaction with NLRP3

**Fig. 4:**
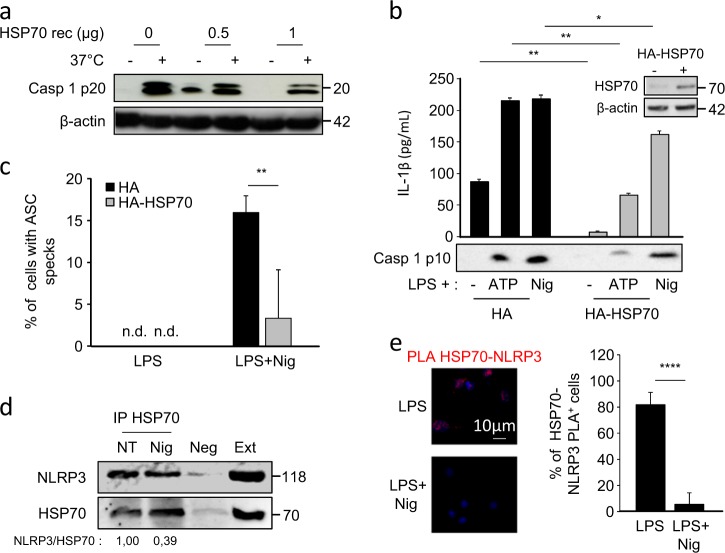
HSP70 inhibits NLRP3 inflammasome through direct interaction with NLRP3. **a** Cell lysates from LPS stimulated THP-1 cells (10 ng/mL) were supplemented with recombinant HSP70 and incubated at 37 °C (+) or left at 4 °C (−). Caspase-1 p20 cleavage fragment was detected by western blot. β-Actin was used as a loading control. **b**–**c** BMDMs from WT C57BL/6 mice were transfected with control vector (HA) or with HA-HSP70, stimulated with 100 ng/mL LPS for 20 h and then left untreated or treated 30 min with ATP (5 mM) or nigericin (Nig, 40 µM). **b** Cell lysates were immunoblotted to detect HSP70 expression (inset). β-Actin was used as a loading control. IL-1β was measured by ELISA and caspase-1 p10 cleavage fragment was detected by western blot in the supernatants. **c** Cells were stained with ASC and NLRP3 antibodies and the proportion of cells with ASC/NLRP3 specks vas evaluated. n.d.: not detected. **d** LPS primed THP-1 cells treated or not with nigericin (30 min, 10 µM) were lysed, immunoprecipitated with anti-HSP70 antibody and analysed by western blot. Neg: lysates without anti-HSP70. Ext: whole cell extracts. Numbers represent NLRP3 expression normalized to immunoprecipitated HSP70 (fold increase), mean of two independent experiments. **e** LPS primed BMDMs treated or not with nigericin (30 min, 10 µM) were stained with anti-NLRP3 and anti-HSP70 antibodies and assayed for PLA. Representative images are shown and the percentages of cells with fluorescent dots were evaluated. Data are the mean of at least three independent experiments ± s.d. Statistics compare HSP70 overexpressing BMDMs with HA transfected cells (**b** and **c**) or untreated with treated BMDMs (**e**): *p < 0.05; **p < 0.01 using two tailed *t* test

Then we checked the effects of HSP70 overexpression on the activation of NLRP3 inflammasome. An in vitro caspase-1 activation assay was first used^10^. Lysates from LPS primed THP-1 human monocytic cells were submitted to 37 °C to activate caspase-1. In this assay, the addition of recombinant HSP70 inhibited caspase-1 activation (Fig. 4a). Second, HA-tagged HSP70 transfected in murine BMDMs triggered a decrease in ATP and nigericin-induced caspase-1 activation and IL-1β maturation, as compared to HA-empty vector transfected cells (Fig. 4b). Finally, HSP70 overexpression inhibited the formation of NLRP3/ASC specks under nigericin treatments (Fig. 4c). As many regulators of the inflammasome act via a direct interaction with NLRP3, we tested whether a potential interaction between HSP70 and NLRP3 existed. Immunoprecipitation in human THP-1 cells and Proximity Ligation Assays (PLA) in murine BMDMs, showed that HSP70 and NLRP3 were associated in LPS-primed cells (Figs. 4d, e). This interaction was reduced in nigericin treated cells (Figs. 4d, e). These results suggest that HSP70 control of NLRP3 inflammasome activation might involve an interaction with NLRP3.

### Heat Shock (HS) inhibits NLRP3 inflammasome activation in vitro and in vivo

**Fig. 5:**
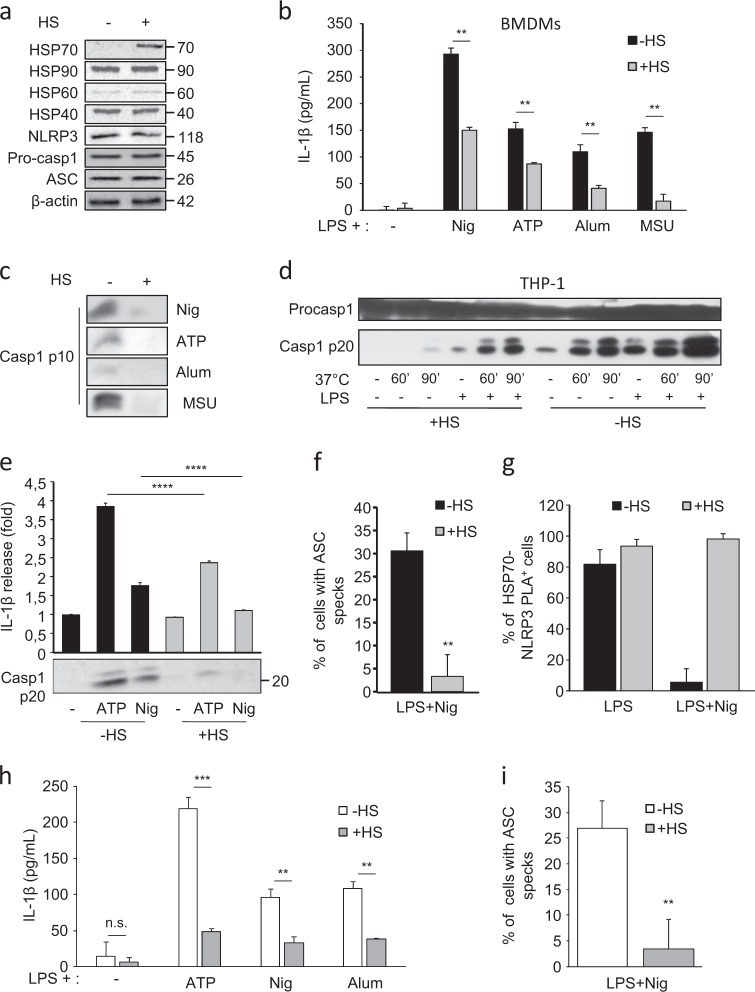
Heat shock inhibits NLRP3 inflammasome activation. Cells were stimulated with LPS (100 ng/mL for BMDMs and 10 ng/mL for THP-1) for 20 h or not when indicated and then submitted or not to a heat shock (HS, 42 °C for 1 h followed by 2 h at 37 °C). **a** BMDMs from WT C57BL/6 mice were lysed and immunoblotted with indicated antibodies. **b** and **c** After ATP (5 mM, 30 min), nigericin (Nig, 40 µM, 30 min), Alum (100 µg/mL, 6 h) or MSU (100 µg/mL, 6 h) treatments, IL-1β was measured by ELISA (**b**) and caspase-1 p10 cleavage fragment was detected by western blot (**c**) in the supernatants of BMDMs. **d** THP-1 cell lysates were incubated at 37 °C or 4 °C, at indicated times. Caspase-1 p20 cleavage fragment was detected by western blot. Procaspase-1 was used as a loading control. **e** After ATP (5 mM, 30 min) or nigericin (Nig, 40 µM, 30 min) treatments, human IL-1β was measured with the HEK-Blue™ IL-1β cells and caspase-1 p20 cleavage fragment was detected by western blot in the supernatants of THP-1 cells. **f** After nigericin (Nig, 40 µM, 30 min) treatments, BMDMs were stained with ASC and NLRP3 antibodies and the proportion of cells with ASC/NLRP3 specks was evaluated. **g** Heat shocked BMDMs treated or not with nigericin (30 min, 10 µM) were stained with anti-NLRP3 and anti-HSP70 antibodies and assayed for PLA and the percentages of cells with fluorescent dots were evaluated. **h** and **i** BMDMs from HSP70^−/−^ C57BL/6 mice were treated as in (**b**) and IL-1β was measured by ELISA in the supernatants (**h**) and the proportion of cells with ASC/NLRP3 specks was evaluated (**i**). Data are the mean of three independent experiments ± s.d. Statistics compare cells with or without heat shock: **p < 0.01; ***p < 0.001; ****p < 0.0001; *n.s.* not significant using two tailed *t* test

**Fig. 6:**
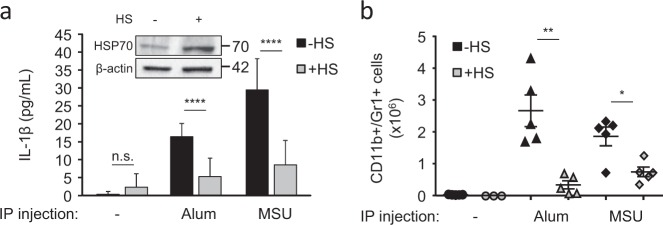
Heat shock inhibits peritonitis in mice. C57BL/6 WT mice (5 animals per group) were heated on a pad (42 °C, 1 h) or not and left 24 h in their cage before being intraperitoneally injected with Alum (40 mg/mL) or MSU (10 mg/mL) for 6 h. IL-1β content in the lavage fluid was measured by ELISA (**a**) and the absolute number of neutrophils (CD11b/Gr1+) recruited was evaluated by flow cytometry (**b**). Insert: a representative western blot of HSP70 expression in peritoneal macrophages in mice. Data also represents the mean ± s.e.m. of three independent experiments. Statistics compare animals with or without heat shock: *p < 0.05; **p < 0.01; ****p < 0.0001; *n.s.* not significant using two tailed *t* test

HSP70 is probably the most universally induced protein after a heat shock. Therefore, to overexpress HSP70, murine BMDMs or human THP-1 cells were submitted to a Heat Shock (HS—42 °C for 1 h followed by 2 h at 37 °C). These conditions were optimal for HSP70 induction, as only the expression of HSP70 was increased among the HSPs studied (Fig. 5a and Supplementary Figure 8). In order to determine the impact of hyperthermia on NLRP3 inflammasome activation, we first checked that the expression of the main components of NLRP3 inflammasome was not modified by the HS in murine BMDMs and human THP-1 cells (Fig. 5a and Supplementary Figure 8). While murine BMDMs primed with LPS and exposed to NLRP3 inflammasome activators, such as nigericin, ATP, Alum or MSU released active caspase-1 and IL-1β in the supernatant, these events were inhibited in cells previously subjected to a HS (Figs. 5b, c and Supplementary Figure 9). In contrast, the HS had no effect on TNFα production (Supplementary Figure 10). These results were also confirmed in THP-1 cells. First, we used the in vitro caspase-1 activation assay. Caspase-1 was activated in cell lysates from non-primed or LPS-primed THP-1 cells submitted to 37 °C. However, when lysates were prepared from THP-1 previously subjected to a HS, caspase-1 activation was reduced (Fig. 5d). Moreover, THP-1 cells primed with LPS and treated with ATP or nigericin released less active caspase-1 and IL-1β in the supernatant when preliminary exposed to a HS (Fig. 5e). Moreover, HS also inhibited the formation of NLRP3/ASC specks (Fig. 5f) and maintained the interaction of HSP70 with NLRP3 (Fig. 5g) under nigericin treatment. We also performed hyperthermia experiments on HSP70^−/−^ macrophages to evaluate the importance of HSP70 in the heat shock-mediated NLRP3 inflammasome inhibition. The heat shock was also able to inhibit IL-1β secretion induced by ATP, nigericin or MSU by HSP70^−/−^ BMDMs (Fig. 5h) and ASC specks formation induced by nigericin (Fig. 5i). Finally, the role of HS in the regulation of NLRP3 inflammasome was evaluated in vivo. Mice were first heated on a pad or not and then injected intraperitoneally with Alum or MSU. Interestingly, neutrophil recruitment and IL-1β maturation were both inhibited when mice were previously subjected to hyperthermia (Figs. 6a, b). Collectively these results show that hyperthermia can regulate NLRP3 inflammasome activation both in vivo and in vitro.

## Discussion

We show here that HSP70 is essential in the regulation of NLRP3 inflammasome activation. Absence of HSP70 triggers NLRP3 inflammasome hyperactivation, leading to a more important caspase-1 activation and IL-1β maturation. Conversely, overexpression of HSP70 inhibits these events. HSP70 inhibitory role may partly be due to its interaction with NLRP3. Finally, as these results were reproduced by a heat shock, we demonstrate that hyperthermia might be an interesting strategy to inhibit NLRP3 inflammasome activation in vivo.

HSP70 joins the family of anti-apoptotic proteins that regulate inflammasome activation such as Bcl-2, Bcl-XL, cIAP1/2, or HSP90^17,22,23^. HSP90 has an ambivalent role in inflammasome activation regulation: while via its association with SGT1, it interacts with NLRP3 and maintains it in an inactive but signal competent state; inhibition of HSP90 activity leads to inflammasome activation impairment^17^. The role of HSP70 seems to be clearer as its knock-down leads to an over-activation of NLRP3 inflammasome, while its overexpression leads to the inhibition of this complex.

HSP70 has been previously described to affect inflammation^24^. First, extracellular or exosome-bound HSP70 can bind TLR2 or TLR4 thus leading to NF-κB activation and TNFα, IL-1β and IL-6 production^25,26^. Second, an anti-inflammatory role for intracellular HSP70 was proposed as it can inhibit LPS-mediated NF-κB activation, through interaction with TRAF6 (TNF Receptor Associated Factor 6)^27^. We propose here another mechanism to explain the ability of HSP70 to regulate inflammation and more particularly NLRP3 inflammasome independently of the NF-κB pathway. Indeed, the lack of HSP70 did not modify the LPS-induced expression of NLRP3 and pro-IL-1β. Moreover, HSP70 expression impacts NLRP3/ASC speck formation probably through its association with NLRP3. Indeed the association of HSP70 with NLRP3 can have an impact on its conformation (because of its chaperone activity) or can block the accessibility of other partners necessary for the formation of NLRP3 inflammasome formation. This will require further investigations.

The capacity of Hsp70^−/−^ cells to produce a high amount of IL-1β is associated with more and larger ASC specks. We can hypothesize that in the absence of HSP70, NLRP3 inflammasome assembly is deregulated under activator treatment and that ASC can oligomerize into several and huge filaments. Maybe the chaperone activity of HSP70 is responsible for the maintenance of one ASC filament formation per cell with a restricted size. Further crystallography experiments should be conducted to evaluate this hypothesis. Such huge ASC complexes were previously described (mean 8 µm, 32 µm max) in cells transfected with ASC D130R mutant with or without ASC full length. However these big ASC specks do not seem to be correlated neither with caspase-1 activation nor with IL-1β maturation^28^. To our knowledge this is the first time that huge specks and/or several specks per cell were observed in a physiological context. Therefore it is surprising that LPS treated Hsp70^−/−^ cells have ASC/NLRP3 and ASC/procaspase-1 oligomers without any caspase-1 activation or IL-1β production. One can speculate that in absence of HSP70, LPS can engage NLRP3 inflammasome assembly and that the second signal such as ATP, nigericin or MSU through K^+^ efflux, ROS generation or cathepsin B release from the lysosomes to the cytoplasm is required to lead to caspase-1 activation. Thus HSP70 would function as a guardian of NLRP3 inflammasome formation.

Furthermore, we propose heat shock as a new non-chemical tool to increase HSP70 expression and inhibit NLRP3 inflammasome activation. However we observed that heat shock was also able to inhibit IL-1β production and ASC specks formation in HSP70^−/−^ BMDMs, suggesting that HSP70 induction is not the only pathway used by hyperthermia to inhibit NLRP3 inflammasome. Several explanations can be proposed. The first one is a compensatory effect induced by other HSPs. The second one can be the involvement of the Heat Shock Factor 1 (HSF-1) which is mainly responsible for the induction of HSP70 expression after a heat shock. Actually, HSF-1 was described to inhibit NLRP3 inflammasome activation through a β-catenin dependent mechanism in murine liver cells^29^, suggesting that in our HSP70^−/−^ model, HSF-1 can compensate HSP70 absence and inhibit inflammation.

Principles of therapeutic hyperthermia on a part or on the whole body were previously described^30^. It could be used to treat patients presenting gain-of-function mutations in genes coding for these complex constituents, e.g., NLRP3 and suffering from periodic fever syndromes, such as cryopyrin associated periodic syndromes^14,31^. It could also be used to treat many diseases associated with a dysregulation of NLRP3 inflammasome such as gout, type 2 diabetes, atherosclerosis, age-related macular degeneration, Alzheimer’s disease or infectious diseases^32^. The ability of HSP70 to inhibit inflammation is in agreement with the results obtained in a study reporting that aggressively treating fever in critically sceptic patients may lead to a higher mortality rate^33^. Fever is one of the most frequent disease symptoms and it can be induced by many cellular mediators. The “pyrogenic cytokine” IL-1β is one of the most important of these mediators^15^. Even if inflammation is well-known to induce fever, contradictory effects of fever or hyperthermia on inflammation were described^34–39^. Here we propose that fever can act as a feedback loop to dampen IL-1β mediated inflammation. This is an important observation since it can help clinicians to decide whether fever must be considered as a friend or as a foe.

In humans, several polymorphisms on genes coding for HSP70 have been reported. Among them, we can notice the HSP70.2 + 1267 G/A polymorphism which was correlated with a lower expression of HSP70 protein in peripheral blood mononuclear cells from multiple sclerosis patients^40^. Moreover, this polymorphism was associated with a higher risk of developing inflammatory diseases, such as Crohn disease, multiple sclerosis or systemic erythematosus lupus^40–42^. Further work is needed to understand how HSP70 can influence the development of inflammatory diseases due to NLRP3 inflammasome dysregulation^32,43^.

In summary, we demonstrate here that HSP70 interacts with NLRP3 and is a new negative regulator of NLRP3 inflammasome activation in vitro and in vivo. Thus, this work proposes a new role for the highly-studied HSP70 and opens avenues for further studies to treat inflammatory diseases, e.g., using hyperthermia.

## Supplementary information

Supplementary methods

Supplementary figures
