# Supplementary material for: HSP70 is a negative regulator of NLRP3 inflammasome activation
Source: Cell Death Dis. 2019 Mar 15;10(4):256. doi: 10.1038/s41419-019-1491-7 (PMC6420651; doi:10.1038/s41419-019-1491-7)
Supplement: Supplementary file 1 — Supplementary methods [file 41419_2019_1491_MOESM1_ESM.docx]

**SUPPLEMENTARY MATERIALS AND METHODS**

**TNFα detection**

Murine TNFα was detected using the BD Bioscience (555268) kit according to manufacturer’s instructions.

**Immunofluorescence (IF)**

The following additional antibody was used: anti-procaspase-1 (1/500, AG-20B-0044, Adipogen).

**Viability assay**

After treatment, cells were washed two times with PBS and fixed with 100% ethanol for 30 min before crystal violet staining. Crystal violet was then resuspended in 33% acetic acid and OD was read at 590 nm with a Wallac 2 spectophotometer (Perkin-Elmer, Villebon sur Yvette, France).

**QPCR**

Total RNA was extracted using Trizol (Invitrogen). One hundred to 300 ng of RNA was reverse-transcribed into cDNA using M-MLV reverse transcriptase, Random Primers and RNAseOUT inhibitor (Invitrogen). cDNA were quantified by real time PCR using Power SYBR^®^ Green Real-time PCR kit (Life Technologies) on a Fast7500 detection system (Applied biosystems). Relative mRNA levels were determined using the ΔΔCt method. Values were expressed relative to actin levels. The following oligonucleotides were used for the detection of *Nlrp3* 5’-acacgagtcctggtgactttg-3’ and 5’-ggcttaggtccacacagaaagtt-3’, *Il1b* 5’-gccaccttttgacagtgatgag-3’ and 5’-agcttctccacagccacaat-3’, or Actin-b 5’-atggaggggaatacagccc-3’ and 5’-ttctttgcagctccttcgtt-3’.
