# Supplementary material for: HSP70 is a negative regulator of NLRP3 inflammasome activation
Source: Cell Death Dis. 2019 Mar 15;10(4):256. doi: 10.1038/s41419-019-1491-7 (PMC6420651; doi:10.1038/s41419-019-1491-7)
Supplement: Supplementary file 2 — Supplementary figures [file 41419_2019_1491_MOESM2_ESM.pptx]

## Slide 1
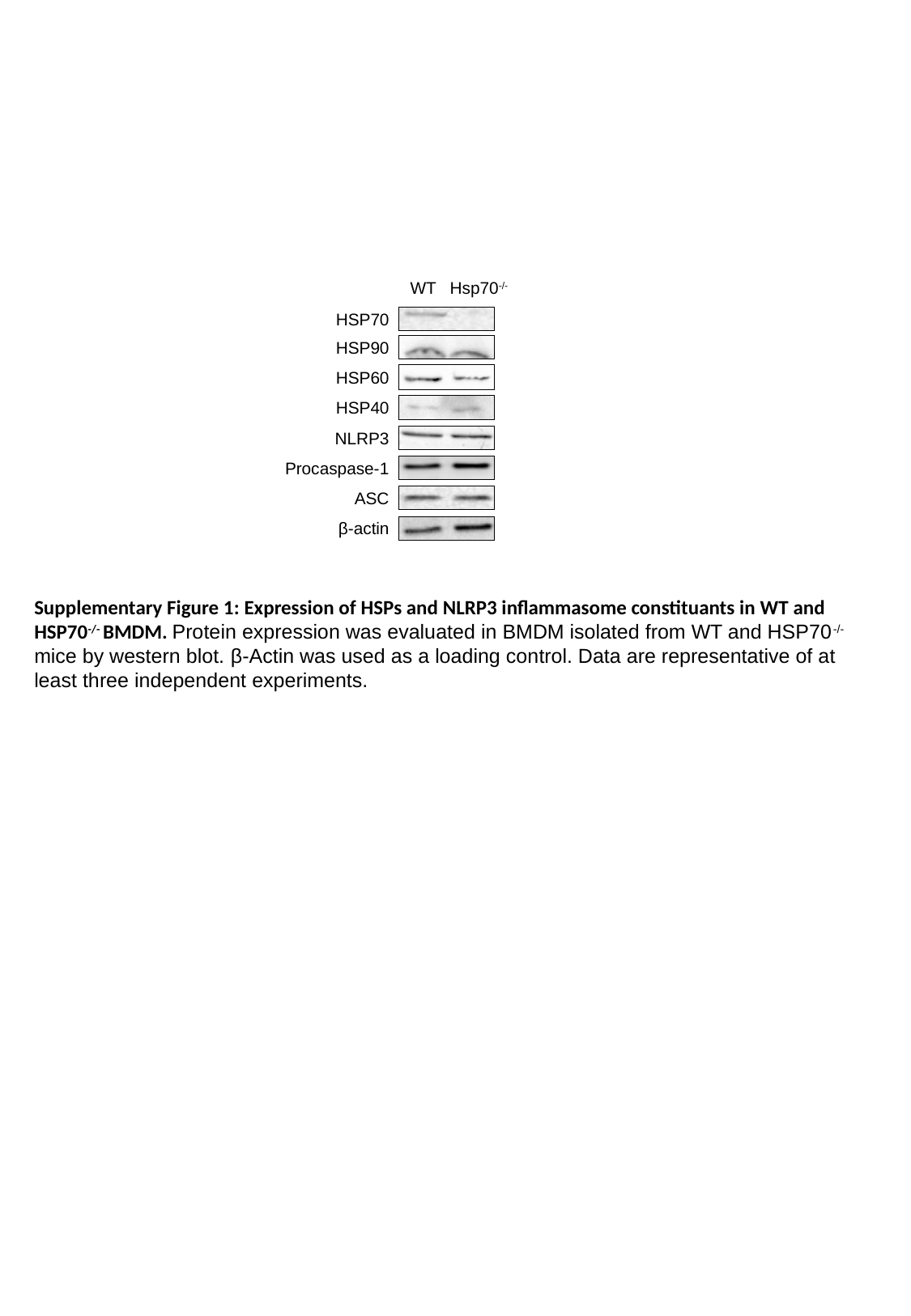

WT Hsp70-/-
HSP70
HSP90
HSP60
HSP40
NLRP3
Procaspase-1
ASC
β-actin
Supplementary Figure 1: Expression of HSPs and NLRP3 inflammasome constituants in WT and HSP70-/- BMDM. Protein expression was evaluated in BMDM isolated from WT and HSP70-/- mice by western blot. β-Actin was used as a loading control. Data are representative of at least three independent experiments.

## Slide 2
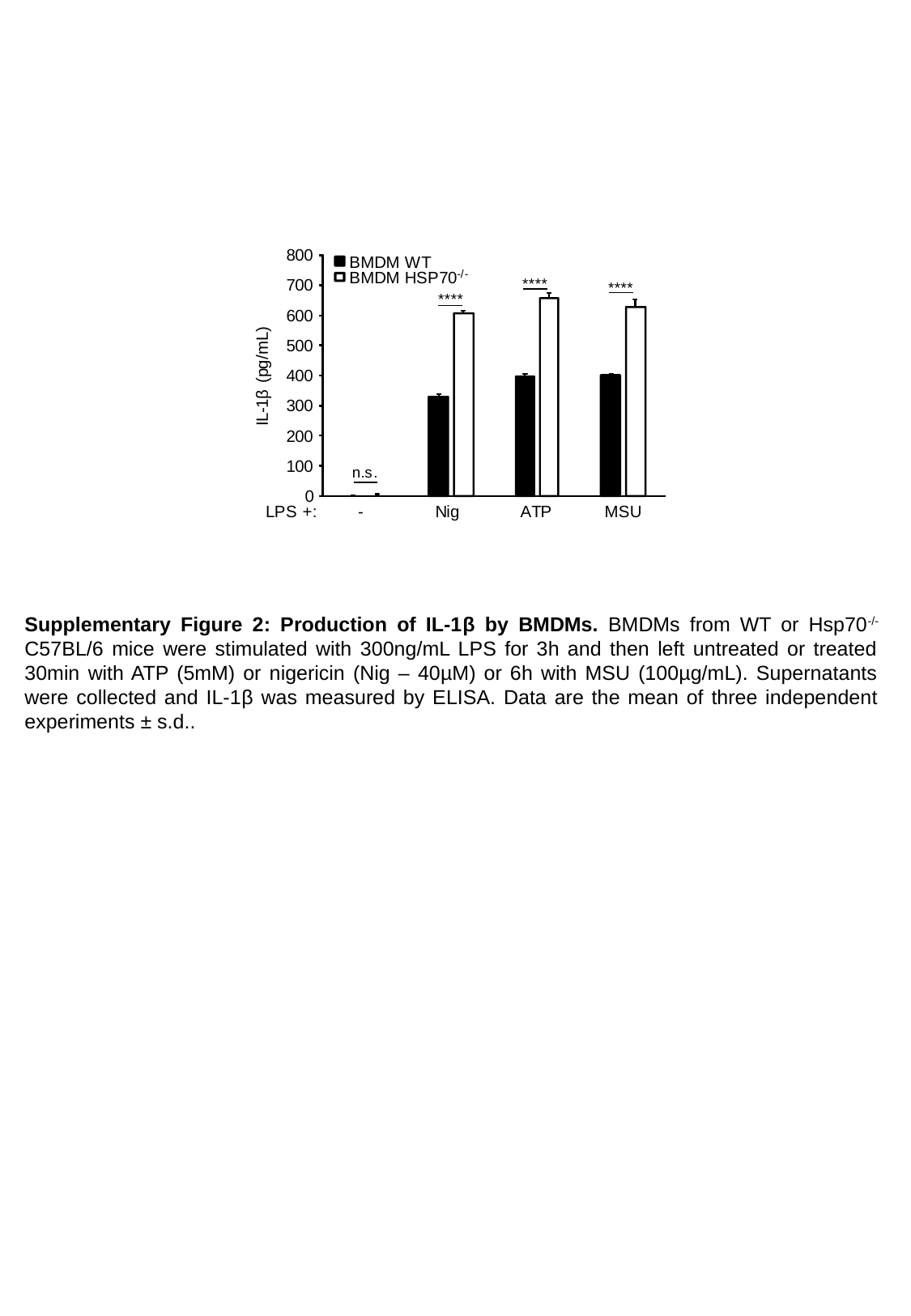

Supplementary Figure 2: Production of IL-1β by BMDMs. BMDMs from WT or Hsp70-/- C57BL/6 mice were stimulated with 300ng/mL LPS for 3h and then left untreated or treated 30min with ATP (5mM) or nigericin (Nig – 40µM) or 6h with MSU (100µg/mL). Supernatants were collected and IL-1β was measured by ELISA. Data are the mean of three independent experiments ± s.d..

## Slide 3
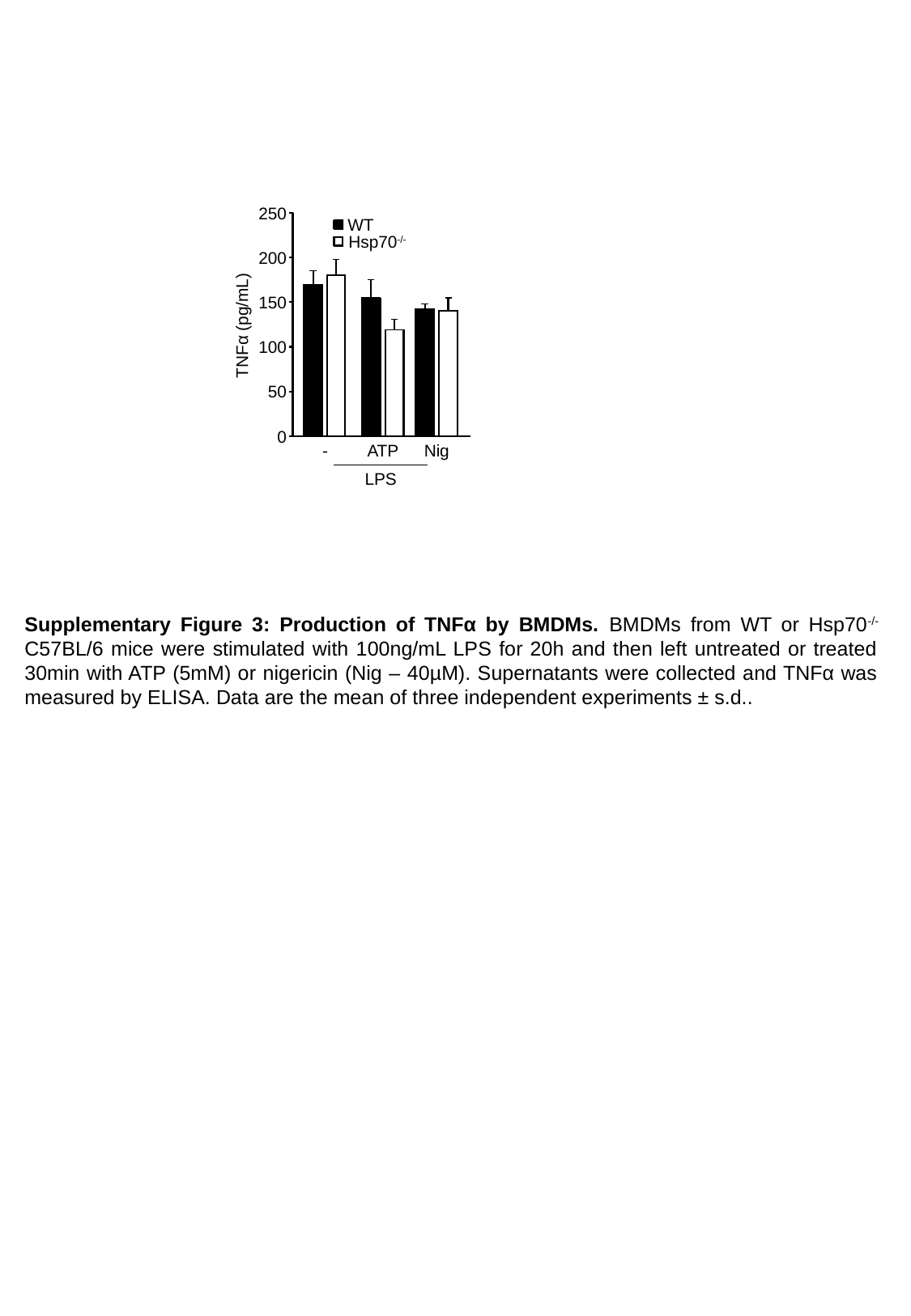

250
WT
Hsp70-/-
200
150
TNFα (pg/mL)
100
50
0
-
ATP
Nig
LPS
Supplementary Figure 3: Production of TNFα by BMDMs. BMDMs from WT or Hsp70-/- C57BL/6 mice were stimulated with 100ng/mL LPS for 20h and then left untreated or treated 30min with ATP (5mM) or nigericin (Nig – 40µM). Supernatants were collected and TNFα was measured by ELISA. Data are the mean of three independent experiments ± s.d..

## Slide 4
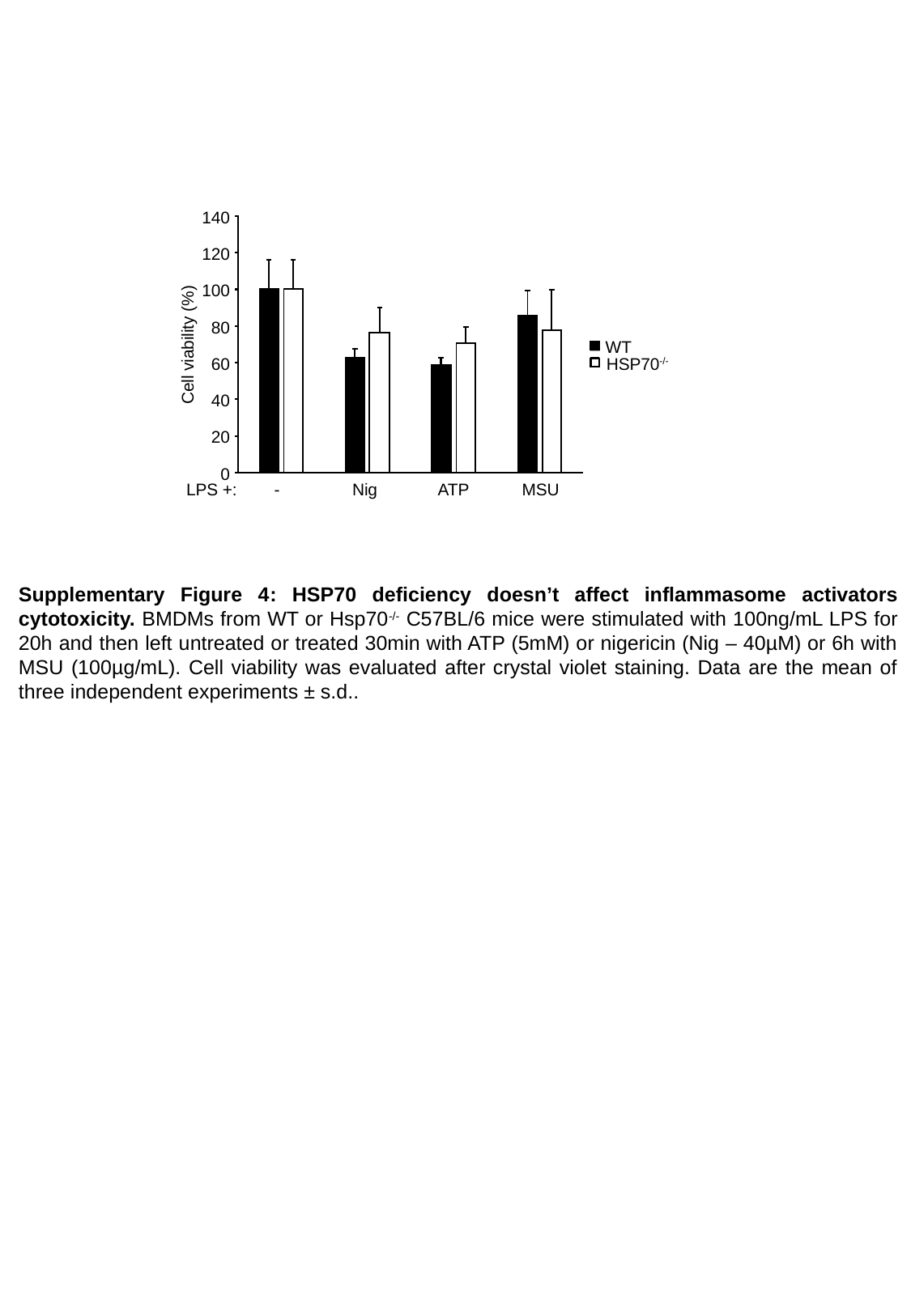

140
120
100
80
Cell viability (%)
WT
HSP70-/-
60
40
20
0
-
Nig
ATP
MSU
LPS +:
Supplementary Figure 4: HSP70 deficiency doesn’t affect inflammasome activators cytotoxicity. BMDMs from WT or Hsp70-/- C57BL/6 mice were stimulated with 100ng/mL LPS for 20h and then left untreated or treated 30min with ATP (5mM) or nigericin (Nig – 40µM) or 6h with MSU (100µg/mL). Cell viability was evaluated after crystal violet staining. Data are the mean of three independent experiments ± s.d..

## Slide 5
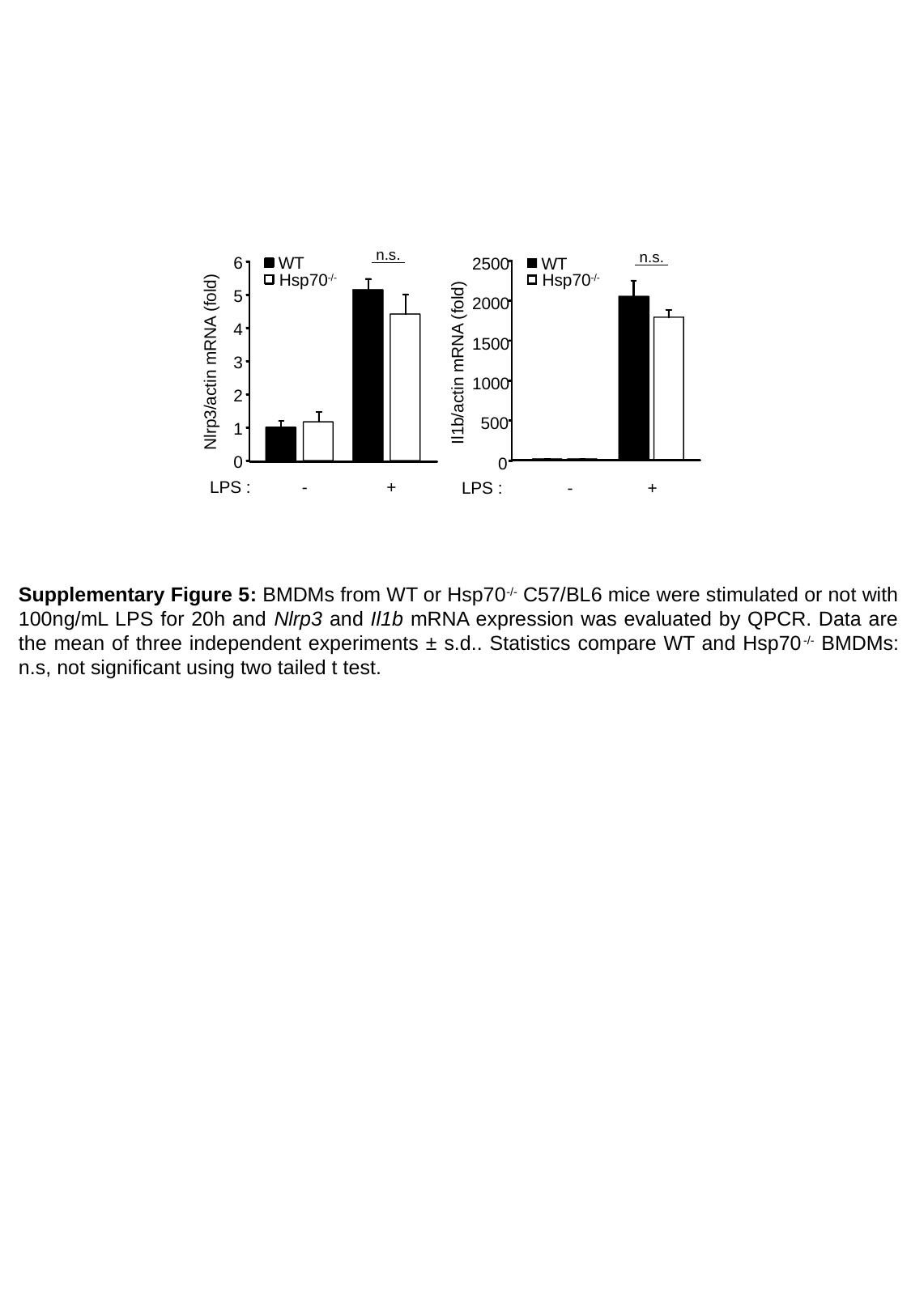

n.s.
n.s.
6
WT
WT
2500
Hsp70-/-
Hsp70-/-
5
2000
4
1500
3
Nlrp3/actin mRNA (fold)
Il1b/actin mRNA (fold)
1000
2
500
1
0
0
LPS : - +
LPS : - +
Supplementary Figure 5: BMDMs from WT or Hsp70-/- C57/BL6 mice were stimulated or not with 100ng/mL LPS for 20h and Nlrp3 and Il1b mRNA expression was evaluated by QPCR. Data are the mean of three independent experiments ± s.d.. Statistics compare WT and Hsp70-/- BMDMs: n.s, not significant using two tailed t test.

## Slide 6
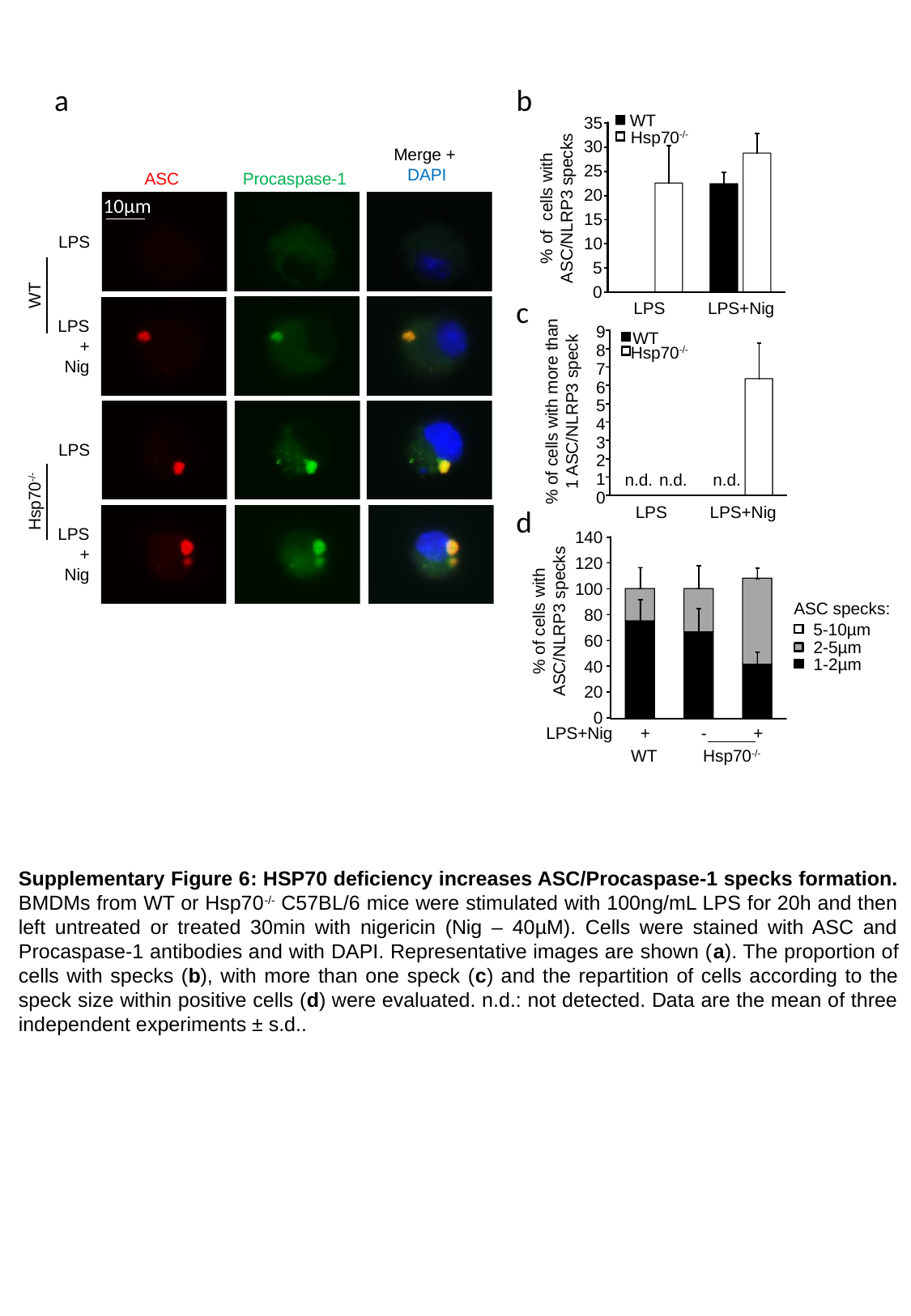

a
b
WT
35
30
25
20
15
10
5
0
Hsp70-/-
% of cells with ASC/NLRP3 specks
LPS
LPS+Nig
Merge +
DAPI
ASC
Procaspase-1
10µm
LPS
WT
c
LPS
+
Nig
9
8
7
6
5
4
3
2
1
0
WT
Hsp70-/-
% of cells with more than 1 ASC/NLRP3 speck
LPS
LPS+Nig
LPS
n.d.
n.d.
n.d.
Hsp70-/-
d
LPS
+
Nig
140
120
100
ASC specks:
% of cells with ASC/NLRP3 specks
80
5-10µm
60
2-5µm
1-2µm
40
20
0
LPS+Nig + - +
WT
Hsp70-/-
Supplementary Figure 6: HSP70 deficiency increases ASC/Procaspase-1 specks formation. BMDMs from WT or Hsp70-/- C57BL/6 mice were stimulated with 100ng/mL LPS for 20h and then left untreated or treated 30min with nigericin (Nig – 40µM). Cells were stained with ASC and Procaspase-1 antibodies and with DAPI. Representative images are shown (a). The proportion of cells with specks (b), with more than one speck (c) and the repartition of cells according to the speck size within positive cells (d) were evaluated. n.d.: not detected. Data are the mean of three independent experiments ± s.d..

## Slide 7
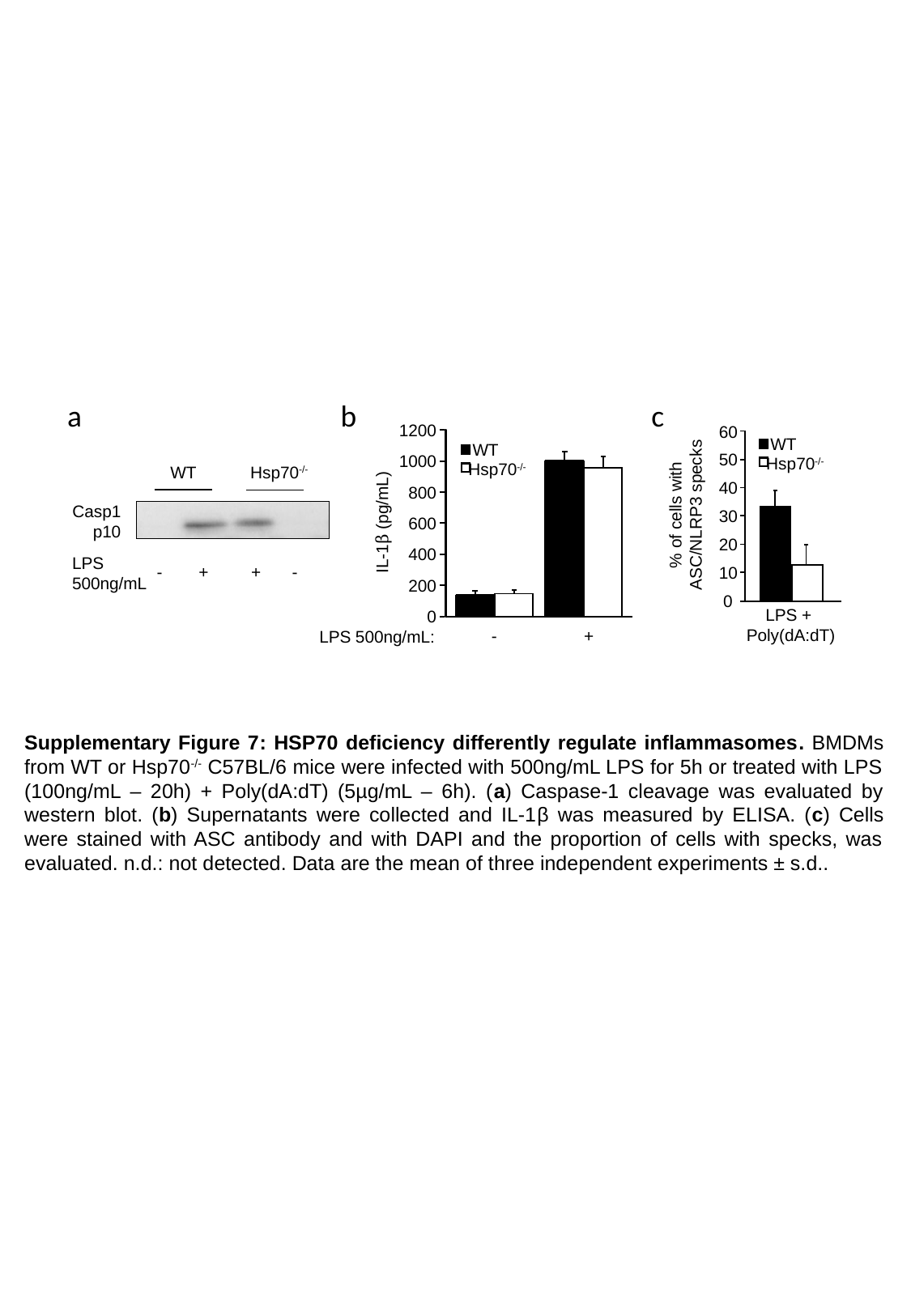

a
b
c
1200
60
WT
WT
50
1000
Hsp70-/-
WT
Hsp70-/-
Hsp70-/-
40
800
% of cells with ASC/NLRP3 specks
Casp1
 p10
30
IL-1β (pg/mL)
600
20
400
LPS
500ng/mL
-
+
+
-
10
200
0
LPS +
Poly(dA:dT)
0
 -
 +
 LPS 500ng/mL:
Supplementary Figure 7: HSP70 deficiency differently regulate inflammasomes. BMDMs from WT or Hsp70-/- C57BL/6 mice were infected with 500ng/mL LPS for 5h or treated with LPS (100ng/mL – 20h) + Poly(dA:dT) (5µg/mL – 6h). (a) Caspase-1 cleavage was evaluated by western blot. (b) Supernatants were collected and IL-1β was measured by ELISA. (c) Cells were stained with ASC antibody and with DAPI and the proportion of cells with specks, was evaluated. n.d.: not detected. Data are the mean of three independent experiments ± s.d..

## Slide 8
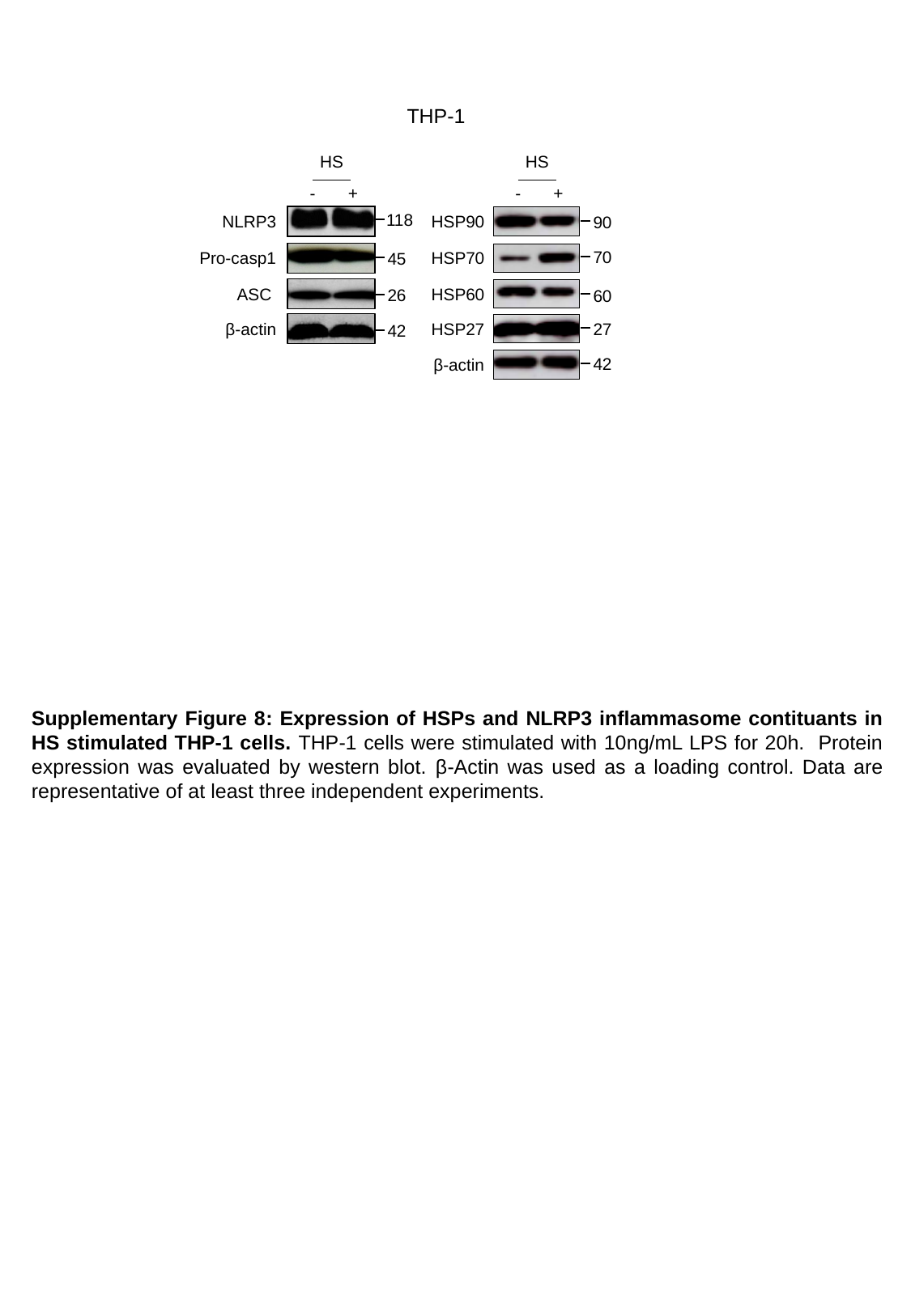

THP-1
HS
HS
 - +
 - +
118
NLRP3
HSP90
90
70
HSP70
Pro-casp1
45
ASC
HSP60
26
60
27
β-actin
HSP27
42
42
β-actin
Supplementary Figure 8: Expression of HSPs and NLRP3 inflammasome contituants in HS stimulated THP-1 cells. THP-1 cells were stimulated with 10ng/mL LPS for 20h. Protein expression was evaluated by western blot. β-Actin was used as a loading control. Data are representative of at least three independent experiments.

## Slide 9
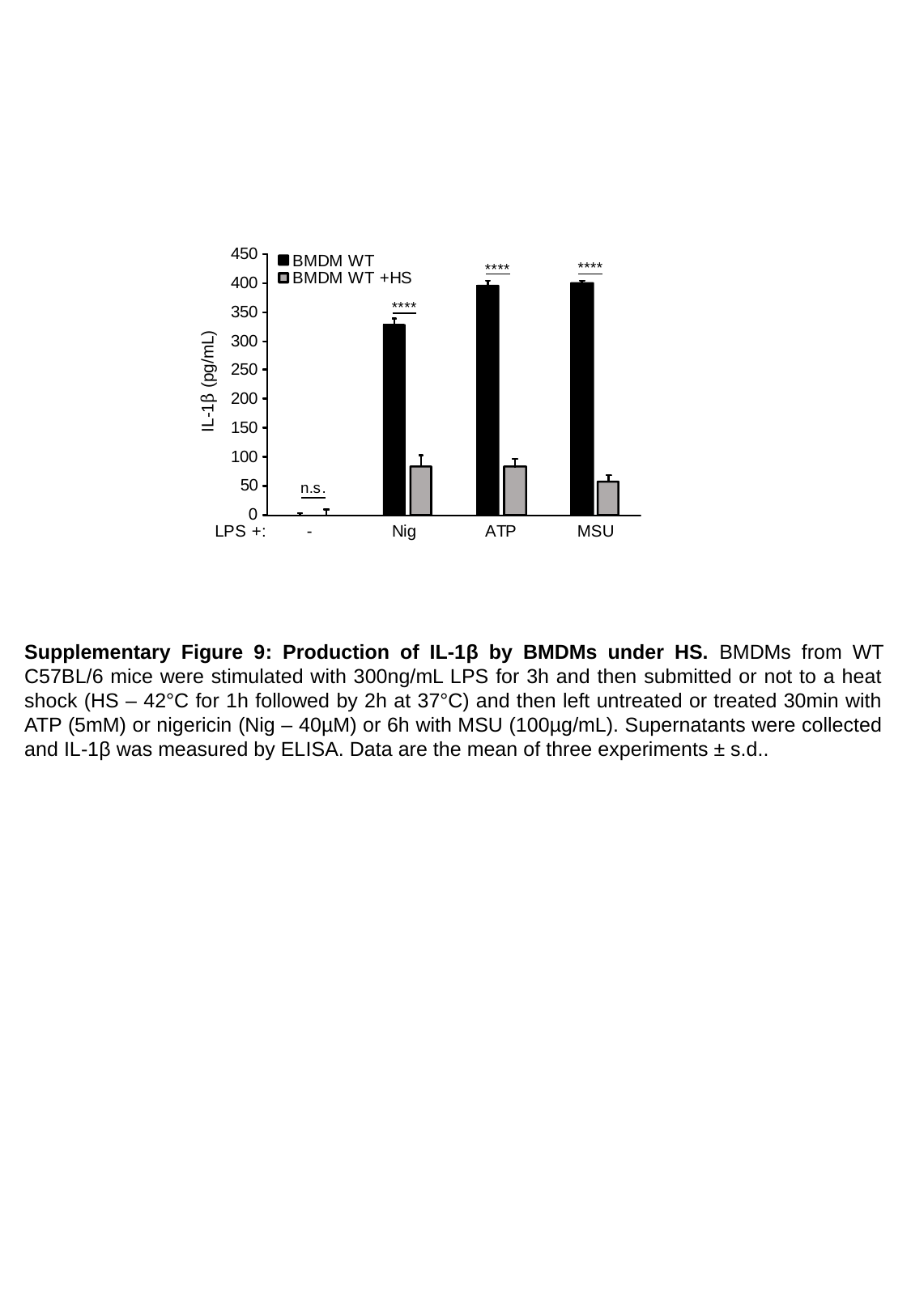

IL-1β (pg/mL)
Supplementary Figure 9: Production of IL-1β by BMDMs under HS. BMDMs from WT C57BL/6 mice were stimulated with 300ng/mL LPS for 3h and then submitted or not to a heat shock (HS – 42°C for 1h followed by 2h at 37°C) and then left untreated or treated 30min with ATP (5mM) or nigericin (Nig – 40µM) or 6h with MSU (100µg/mL). Supernatants were collected and IL-1β was measured by ELISA. Data are the mean of three experiments ± s.d..

## Slide 10
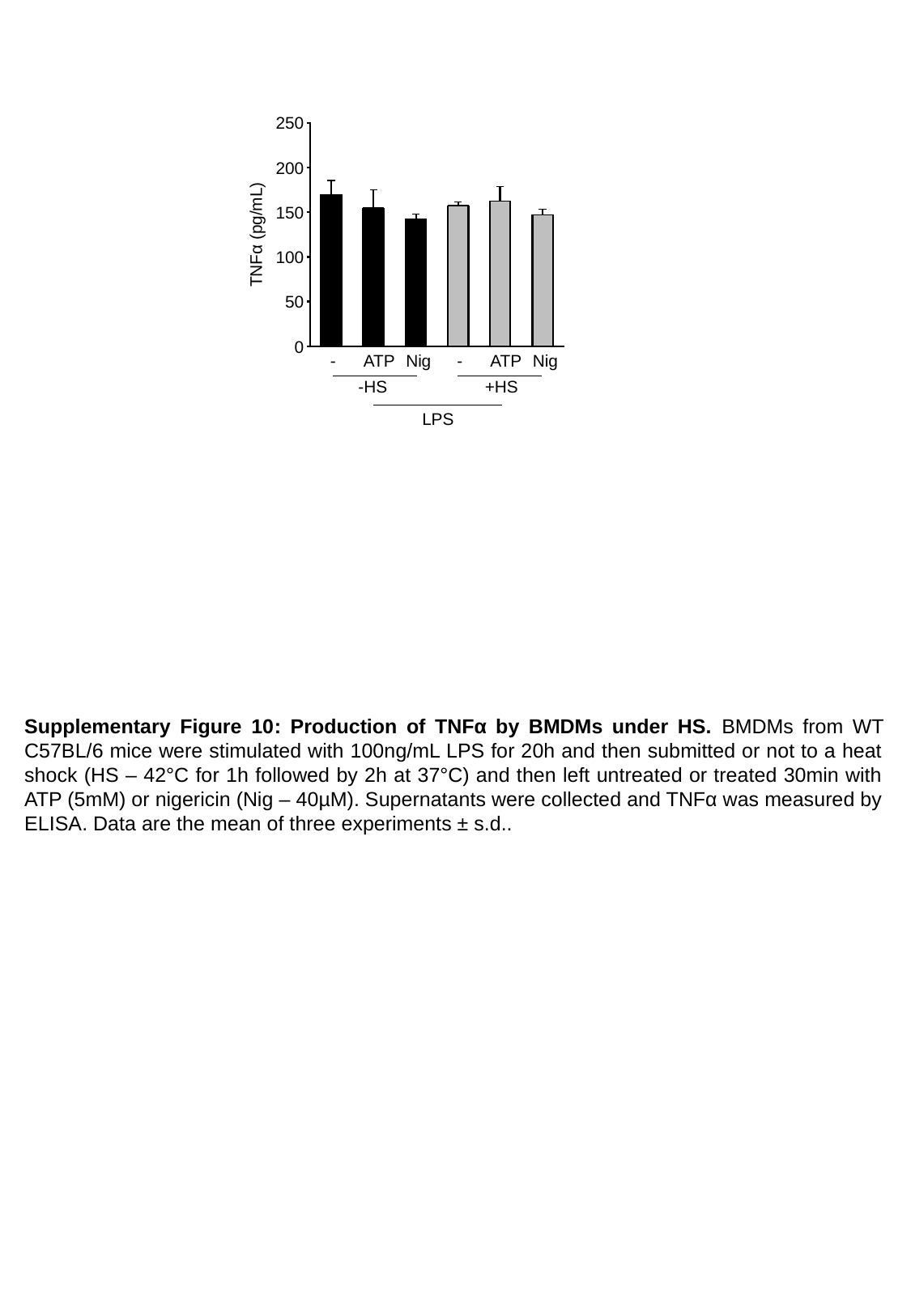

250
200
150
TNFα (pg/mL)
100
50
0
-
ATP
Nig
-
ATP
Nig
-HS
+HS
LPS
Supplementary Figure 10: Production of TNFα by BMDMs under HS. BMDMs from WT C57BL/6 mice were stimulated with 100ng/mL LPS for 20h and then submitted or not to a heat shock (HS – 42°C for 1h followed by 2h at 37°C) and then left untreated or treated 30min with ATP (5mM) or nigericin (Nig – 40µM). Supernatants were collected and TNFα was measured by ELISA. Data are the mean of three experiments ± s.d..
